# Retraction: High glucose induced HIF-1α/TREK1 expression and myometrium relaxation during pregnancy

**DOI:** 10.3389/fendo.2025.1589452

**Published:** 2025-03-14

**Authors:** Frontiers Editorial Office

The journal retracts the February 27 2023 article cited above.

Following publication, concerns were raised regarding the integrity of the images in the published figures. The authors failed to provide a satisfactory explanation during the investigation, which was conducted in accordance with Frontiers’ policies. As a result, the data and conclusions of the article have been deemed unreliable and the article has been retracted. The authors do not agree to this retraction.

